# Wear Behavior of Different Generations of Zirconia: Present Literature

**DOI:** 10.1155/2022/9341616

**Published:** 2022-03-07

**Authors:** Kuljirarnat Jitwirachot, Pimduen Rungsiyakull, Julie A. Holloway, Wissanee Jia-mahasap

**Affiliations:** ^1^Department of Prosthodontics, Faculty of Dentistry, Chiang Mai University, Chiang Mai, Thailand; ^2^Department of Prosthodontics, The University of Iowa, Iowa City, IA, USA

## Abstract

**Objective:**

The wear behavior of the novel zirconia generation is less well understood and may be affected by compositional modifications compared to the conventional zirconia.

**Materials and Methods:**

Combinations of keywords such as “zirconia,” “high translucent,” and “wear” were searched in PubMed and Google Scholar databases up to May 2021. The total of 23 relevant articles was selected according to inclusion criteria.

**Results:**

Reports show comparable wear resistance of translucent zirconia to the conventional zirconia despite an increased cubic phase content and lower mean flexural strength. A meticulously polished surface creates the lowest surface roughness, producing favorable zirconia wear resistance and antagonist wear compared to a glazed surface. In comparison to other ceramic materials, zirconia produces the least wear on an enamel antagonist and almost undetectable wear when opposed by zirconia. Wear when paired against resin materials yields a favorable outcome, whereas wear behavior against a metal antagonist varies with the surface hardness of the metal.

**Conclusions:**

All zirconia generations are considered wear-friendly to all types of antagonists. Nonetheless, comparative studies on antagonist wear opposing zirconia of different compositions are still limited and further investigation is required.

## 1. Introduction

With a rising demand from patients for highly esthetic dental restorations, various tooth-colored restorative materials have been introduced in the past decades. Zirconia rapidly gained popularity due to several advantages including good esthetics, biocompatibility, and outstanding mechanical properties comparing to other ceramic systems. In addition, increasing prices of precious alloys and mechanical failure of metal-ceramic restorations have made zirconia a novel restoration of choice.

Three generations of zirconia have been developed to date. Initially, conventional 3Y-TZP (3 mol% yttria-stabilized tetragonal zirconia polycrystal) was utilized as a framework material that was veneered by more conventional ceramics to achieve an esthetic result, as this first-generation dental zirconia was very opaque. Its high strength allowed multiunit applications, but chipping and failure of the weaker veneering ceramic were common. The latter two generations, shared termed “high-translucency” zirconia, aim to overcome esthetic problems, namely, high opacity, exhibited by previous generations of zirconia. Improved translucency of zirconia from the second generation was achieved through reduction of alumina additive content to 0.05% by weight while yttrium oxide stabilizer remains unchanged at 3% by mol (4.5–5.6% by weight) [1]. Further translucency is enhanced with rising yttrium oxide content above 4% by mol (<10% by weight) along with the amount of cubic phase zirconia. These modifications lead to plausible application of zirconia as monolithic restoration without the need for veneering. However, while the change in chemical composition to improve the optical properties does not significantly affect the physical and mechanical properties of second-generation zirconia, the modifications adversely lessen those of the third-generation zirconia [1]. Our review will focus on the effects of these compositional changes in the different generations of zirconia on wear resistance.

Investigation of opposing enamel and restoration surface wear is of equal importance. Conventional wisdom, based on wear of feldspathic ceramics opposing natural dentition, has suggested that a new zirconia restoration should not be placed opposing unrestored enamel surfaces. Likewise, interaction of zirconia restoration with other types of restorative material was concerned. Additionally, wear resistance of the zirconia restorations itself is noteworthy. Therefore, our review aims to discuss the wear behavior of zirconia as well as its opposing materials and provide evidence that will be useful for making a clinical decision when selecting the appropriate material for fixed restorations.

**Figure 1:**
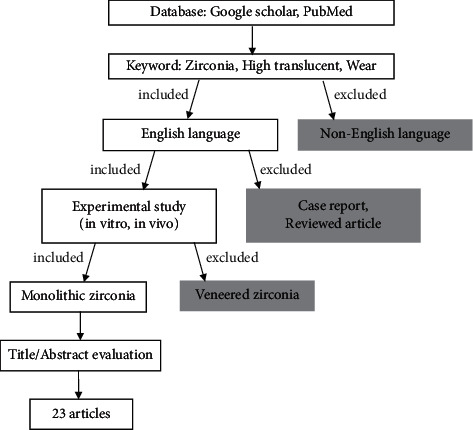
Article selection flowchart.

A literature search of electronic databases was conducted using PubMed and Google Scholar databases up to May 2021. The search keywords included combinations of terms such as “zirconia,” “high-translucent,” and “wear.” Experimental studies, both *in vitro* and *in vivo*, involving wear of monolithic zirconia and its opposing materials of any types were included. The publication must be in English language. Case report and non-peer-reviewed articles were excluded. From all search results appeared on the database, only 23 available articles were selected after analyzing for relevant titles and abstracts. The flowchart of article selection is illustrated in Figure 1.

## 2. Zirconia in Dentistry

Zirconia material is originally known for its superior flexural strength and surface hardness. High surface hardness was expected to produce more antagonist wear [2, 3]. This statement holds true for metals, which wear through plastic deformation [4, 5]. However, evidence suggests a poor correlation between ceramic materials and surface hardness because of their brittle nature [6]. Accordingly, the wear mechanism of ceramics is abrasive wear as a result of surface microfracture [7]. Ability to withstand external force and maintain surface integrity depends on properties of the material. Therefore, understanding the variation of zirconia, both chemically and mechanically, which might have an influence on wear behavior is crucial.

### 2.1. Basic Information

**Figure 2:**
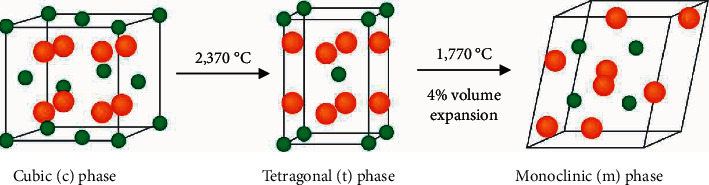
Phase transformation of zirconia.

Zirconia or zirconium dioxide (ZrO_2_) is a polymorphic material existing in three crystalline phases: monoclinic, tetragonal, and cubic (Figure 2). It can undergo phase transformation under the influence of temperature or stress. Without chemical modification, zirconia crystalizes in its cubic phase (*c* phase) during cooling first at temperatures below 2,680°C. It then transforms to the tetragonal phase (*t* phase) at 2,370°C and lastly to the monoclinic phase (*m* phase) under 1,770°C. The latter phase transformation causes volumetric expansion of approximately 4%. Such sudden change in volume creates high tension and undesirable crack formation in the ceramic. Initially, 3 mol% yttria or yttrium oxide was added to zirconia to prevent this phase transformation [1, 8].

**Figure 3:**
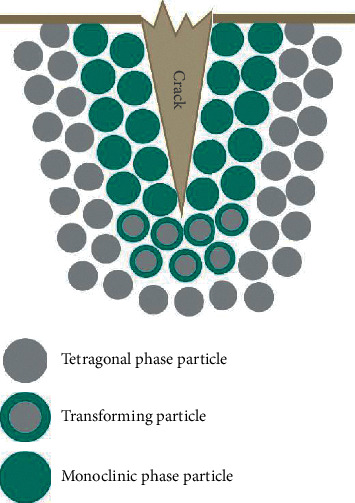
Transformation toughening.

Yttria-stabilized zirconia has a unique property which brings about its high strength. First-generation zirconia is stabilized in the tetragonal phase with 3 mol% yttria preventing it from transforming to the monoclinic phase. However, exposure to mechanical stress and subsequent crack initiation leads to localized phase transformation. The change to monoclinic phase results in volumetric expansion, and a compressive force is produced at the advancing crack front, essentially “pinching” the crack closed. This prevents the crack from propagating and therefore increases the strength of 3Y-TZP. This phenomenon is called transformation toughening (Figure 3). This event may also help maintain surface integrity and smoothness which may reduce antagonist wear [9].

Another phase transformation in zirconia is called low-temperature degradation (LTD) or aging. This phenomenon happens slowly from water penetration into crystalline structure at 200–400°C well below sintering temperature. Repeated exposure to warm and humid environment gradually transforms phases of zirconia from tetragonal to monoclinic. The rate of LTD, stated in a study by Koenig et al. in 2006, is approximately 15 years or comparable to the mean lifespan of dental restorations [10]. Phase transformation from LTD potentially could roughen the surface of zirconia along with the formation of microcrack [11].

An addition of alumina content of at least 0.15 wt% helps reduce low-temperature degradation. Reduction of alumina content to enhance zirconia translucency can increase predisposition to LTD. Nevertheless, alumina sintering additives also lead to higher opacity, a competing outcome for dental use.

### 2.2. Generation of Zirconia

#### 2.2.1. First Generation

The first generation of zirconia was introduced to dentistry over two decades ago as yttria-stabilized tetragonal zirconia polycrystal (Y-TZP). It contains 3%mol of yttria with at least 90 percent of tetragonal zirconia, giving its name, 3Y-TZP. Despite superior mechanical property with flexural strength over 1000 MPa [1, 12], this generation of zirconia has opaque characteristics which limit its use in esthetic areas. Alteration of the sintering protocol was attempted to improve translucency. Unfortunately, it adversely decreased flexural strength and does not succeed [1]. This compromised optical property of conventional zirconia necessitates the use of veneering porcelain over the zirconia framework to mask its opacity [13]. Therefore, as with conventional metal-ceramic restorations, frequent chipping of veneering porcelain was observed and was reported as ranging from 0–54% annually [14].

#### 2.2.2. Second Generation

The second generation of zirconia was proposed to solve the frequent chipping problem as a monolithic or single-layered restoration [15]. Furthermore, in addition to elimination of the unwanted chipping complication, monolithic restorations require less invasive nature, tooth reduction, and less laboratory time and cost to fabricate [16]. However, the main composition is still 3% mol of yttria with at least 90 percent of tetragonal zirconia. Better translucency was achieved by reduction of alumina additive content from 0.25% to 0.05% by weight. Alumina has a large refractive index mismatch with zirconia which leads to light scattering and diminishes zirconia translucency. A reduction in lumina content, as well as alumina molecule rearrangement, allows higher light transmission [17, 18]. These changes brought about the second generation of dental zirconia. It still exhibits comparable flexural strength to the first generation at 1000 MPa, ten times higher than veneering porcelain, without jeopardizing stability and strength of the zirconia [1, 19]. In addition, staining and glazing can enhance esthetics and mimic a natural appearance. However, with only 70% of the translucency of lithium disilicate, the second generation of dental zirconia still did not provide adequate esthetics for use in anterior teeth [13].

#### 2.2.3. Third Generation

The third and the latest generation of zirconia exhibits a modified crystalline structure containing increased percentages of cubic phase in pursuit of even greater translucency. “High-translucent” or “ultratranslucent” zirconia refers to this specific generation. The geometry of different zirconia phases affects light transmission behavior and translucency. The tetragonal phase has birefringent properties, or anisotropic refractory index, which results in a greater amount of light scattering at grain boundaries. On the other hand, the cubic phase has isotropic refractory index without scattering effect which offers better translucency.

Increasing the content of yttria to 4% and 5% mol results in higher nontransformable cubic phase and lesser tetragonal phase, consequently increasing translucency. Elemental analysis through X-ray diffraction (XRD) by Zhang et al. revealed about 40% and 60% of cubic phase content in 4Y- and 5Y-TZP, respectively [20]. Other studies also report an increased amount of cubic phase of up to 53% in this third-generation zirconia (5Y-TZP) [1, 18].

It is noteworthy that there are some confusions on the term “translucent zirconia” since both second and third generations are frequently called by the same nomenclature. The term “monolithic zirconia” without any further description is normally referred to 3Y-TZP from the second generations, while studies on the third generation are usually more specific [21–24]. However, detailed compositions of commercial products are scarcely provided by the manufacturers or researchers. Some available information on manufacturers and compositions was summarized in a review by Kontonasaki et al. [25].

### 2.3. Property Changes in the Third Generation

#### 2.3.1. Optical Properties

Improvement in translucency of 5Y-TZP was evaluated in multiple studies [23, 26, 27]. 5Y-TZP has the highest translucency among other generations of zirconia owing to greater amount of cubic zirconia and its grain size. It appears to occupy a place between lithium disilicate (IPS e.max CAD LT) and conventional 3Y-TZP zirconia, in terms of both translucency and flexural strength [1, 20, 23, 28]. Baldissara et al. conducted a comparative study in 2008 on contrast ratio between lithium disilicate glass ceramic and 5Y-TZP zirconia using an anatomic crown shape with clinically recommended occlusal thickness. The result revealed no significant difference in translucency [29]. Regardless of material color, the translucency of zirconia is less influenced by thickness unlike lithium disilicate [1, 12, 30, 31]. Expected light transmittance percentage of 5Y-TZP as claimed by the manufacturers is still more opaque than average human dentin.

#### 2.3.2. Mechanical Properties

The mechanical properties of high-translucency zirconia are manufacturer-dependent. Even though increased cubic phase enhances translucency, it negatively influences strength of high-translucent zirconia. Cubic zirconia does not possess the transformation toughening phenomenon as does tetragonal zirconia. Less transformation can be detected in 4Y-TZP, but no evidence is shown in 5Y-TZP [20]. The deprivation of this unique property of previous generations leads to a reduction in flexural strength in 5Y-TZP to about one-half to two-thirds of 3Y-TZP [18, 20, 23, 27, 32–35]. Clinical applications might be more suitable for low stress-bearing areas. Although 5Y-TZP exhibits higher fracture resistance compared to lithium disilicate when tested at unbounded state, they demonstrate similar fracture strength when bonded to dentin-like substrate at 0.5 and 1 mm thickness and inferior fracture strength for 5Y-TZP at 1.5 mm thickness [36].

The transformation toughening phenomenon is a unique property which contributes to the high strength of 3Y-TZP zirconia by reducing crack propagation and increasing fracture toughness [1, 9]. This event may also help maintain surface integrity and smoothness which consequently reduces antagonist wear [9]. Without this specific property in 5Y-TZP zirconia, surface integrity and wear resistance may be affected. Conversely, the lack of phase transformation prevents low-temperature degradation [1, 18, 33, 34], and surface smoothness might be thereby compensated. However, concerns about wear resistance of third-generation zirconia should still be noted.

## 3. Wear Behavior of Zirconia

In terms of occlusal wear, ideal restorative materials should possess a similar wear rate to physiological enamel wear rate which ranges from 30 to 40 *µ*m per year in molar area and relatively lesser as the position in arch moves anteriorly [3, 37, 38]. A mismatch in wear rate between opposing teeth may result in excessive wear and lead to impaired esthetics and function [39, 40]. Two aspects of interest in terms of wear have been discussed, namely, wear of the material itself and wear of its antagonist which is affected by material abrasiveness.

### 3.1. Factors Related to Zirconia Wear

Wear mechanism is multifactorial. Physiological variations among patients and material-based factors both play an important role in the wear process. Factors influencing wear of ceramics were thoroughly reviewed by Oh et al. in 2002 including physical factors, microstructural factors, chemical factors, and surface finishing [5].

#### 3.1.1. Physical Factors

Ceramic materials have low resistance to tensile stress due to their brittle nature, which leads to surface chipping or fracture either from physiologic wear or iatrogenic surface adjustment [7]. With its higher fracture toughness, zirconia is less predisposed to crack formation. Also, transformation toughening counteracts crack propagation. Therefore, zirconia has an ability to maintain surface integrity under higher stress compared to other dental ceramics.

Frictional coefficient, a ratio of frictional force between two sliding surfaces in relation to the normal force pressing the two surfaces together, is varied by occlusal anatomical variations, degree of mandibular movement, masticatory load, chewing rate, types of contacting materials, and surrounding environment [5]. Patients with a wider range of movement or parafunctional habits, as well as greater masticatory load and/or sliding velocity, tend to generate a higher frictional load, resulting in greater wear [5, 41]. However, these physiological factors cannot be altered by the dentist.

Irregularities in a ceramic surface contribute to surface roughness which can significantly accentuate antagonist wear in ceramic material itself and the opposing surface [5, 42, 43]. Roughened zirconia surfaces increase not only the wear of the opposing surface but also the incidence of chipping. Surface treatment methods and intraoral occlusal adjustment affect degree of surface roughness. Hence, an optimal smooth surface would benefit long-term reliability [44].

#### 3.1.2. Microstructural Factors

Differences between the microstructures of glass ceramics and polycrystalline ceramics contribute to the difference in their wear behavior and wear mechanism. Glass ceramics are composed of crystalline particles incorporated into glassy matrix. Their principal wear mechanism relies on fatigue fracture of the glassy matrix with consequent exposure and loss of embedded crystalline phase [45]. Variations in base mineral types, amount, configuration, and distribution of these crystals are responsible for the abrasiveness of each ceramic [46]. On the other hand, polycrystalline ceramics exhibit a dense crystalline phase composition with little or no glassy phase. These polycrystalline ceramics are distinguished by high fracture toughness and wear through an abrasive wear mechanism where minor grain dislodgment is observed under scanning electron microscopy but no microcrack or chipping fragments are typically seen [47–51]. Although having similar fracture toughness and strength, lithium disilicate glass and 5Y-TZP display distinctly different wear behaviors due to differences in their microstructure [47].

Besides surface irregularities generated from functional wear, defects and porosities from the manufacturing process also aggravate the wear process on opposing surfaces once subsurface pores are exposed, leading to sharp asperities [7, 52].

#### 3.1.3. Chemical Factors

Variations in pH values affect zirconia in a different way. In one study, low coefficients of friction and wear rates were observed in acidic environments below pH 4. The surface roughness of zirconia was not affected and actually decreased after exposure to acid, owing to a tribocorrosion process [53]. In contrast, a greater coefficient of friction and wear rate were observed in an intermediate to high pH environment. In highly alkaline environments, severe surface fractures and large dislodged fragments were detected, as well as phase transformation induced by low-temperature degradation [53, 54]. However, fluctuation of intraoral pH from normal value about 7–7.3 to higher pH values is uncommon but to lower pH could occur in patients with regurgitation of gastric content, acidic intakes, or mouth breathing [5, 55]. Although zirconia restoration might survive under acidic environment, wear of opposing dental hard tissue or glass ceramics would be aggravated [5]. Under the similar acidic condition, ability to maintain surface integrity of zirconia would be more beneficial to opposing surface compared to increase in surface roughness and abrasiveness of glass ceramic restoration which undergo etching and glass corrosion process [5].

#### 3.1.4. Surface Finishing

Surface finishing is associated with surface roughness and wear of zirconia. Comparison of glazed and polished surfaces has been widely studied. Most of the studies on zirconia surface finishing are in agreement with the polishing methods used to produce the least wear of the zirconia itself and the antagonist [49, 56–62]. Glazed surfaces may appear almost mirror-like at first glance from filling and leveling the rough surface. However, after a short period of time, the thinly applied glaze layer wears off and reveals a rougher, unpolished surface underneath resulting in increased antagonist abrasion [26, 51, 57, 58]. Polishing prior to glazing reduces chance of excessive surface wear [63]. Repolishing after the surface has lost its glaze is also recommended [64]. Moreover, occlusal adjustment using diamond burs significantly roughens the adjusted area and requires proper finishing and polishing afterward [59–61].

Different polishing systems and polishing protocols by researchers produce varying degrees of surface roughness. The grit of diamond bur used for adjustment also correlates to postadjustment surface roughness. Adjustment of zirconia with a fine 30 *µ*m grit bur produces statistically comparable wear of opposing enamel as that of polished zirconia, while adjustment with a coarse 100 *µ*m grit bur generates substantially increased wear [57]. A fine diamond bur produces a surface roughness of approximately 1.18 *µ*m, but for a coarse diamond bur, surface roughness is reported to be as high as 3.95 *µ*m. The threshold value of ceramic surface roughness (Ra) that significantly raises enamel wear based on several studies is over 1.5 *µ*m [57, 65, 66]. Nonetheless, when comparing wear of enamel after zirconia adjustment with a fine 30 *µ*m grit bur vs. zirconia polishing burs, a statistically significant decrease in surface roughness and wear was reported from polishing [63]. Regarding the selection of zirconia polishing systems, diamond-impregnated silicone burs specifically designed for zirconia and other high strength ceramics are more effective in generating a smoother monolithic zirconia surface than silicon carbide-impregnated silicone burs, designed for porcelain polishing [67, 68]. A meticulously polished surface using the appropriate burs should provide a surface roughness as low as 0.1–0.4 *µ*m [56, 63, 69]. Force and friction applied during polishing, although while producing some local heat and stress, neither effect surface roughness nor create stress-induced *t-m* phase transformation [56, 68, 69]. In addition, low surface roughness produced from polishing results in enhanced wear resistance and phase transformation resistance during the wear process of zirconia [56]. Even if differences in surface treatment have a direct effect on wear rate of the opposing surface, all abraded zirconia specimens showed no visible signs of wear regardless of surface treatment protocols [63].

Application of external staining material is another factor promoting wear since the composition of extrinsic stains contains abrasive metal oxides [5]. The surface roughness of zirconia, as well as antagonist wear volume, was reported to be greater after staining [62]. Therefore, its application should be restricted only to the surfaces not in occlusal contact.

### 3.2. Wear Resistance of Zirconia

Studies on various dental restorative materials have stated that materials with high strength and high fracture toughness showed minimal material wear or antagonist wear [5, 21, 50, 51, 58]. Scanning electron microscopy of zirconia also reveals a tightly bound grain structure which resists surface degradation [21]. Previous reviews on the mechanical properties of high-translucent zirconia showed that the higher yttria content in cubic zirconia negatively influences fracture toughness and flexural strength of zirconia [18, 20, 70]. On the other hand, low-temperature degradation which induces surface roughness and subsurface crack formation is less in cubic zirconia than stabilized zirconia compositions [11]. This might consequently affect wear of the materials.

Concerns involving surface stability have led to studies evaluating wear behavior among the different zirconia generations. Most of the previously published studies were conducted with 3Y-TZP, and there is little information on wear behavior of high-translucent zirconia with increased yttria content. However, a few recent studies have compared wear behavior between 3% mol and 4-5% mol yttria-stabilized zirconia [21–24, 47]. 3Y-TZP, 4Y-TZP, and 5Y-TZP zirconia compositions exhibited minimal to unmeasurable wear volume loss regardless the type of antagonists including human enamel, steatite ball, or 3Y-TZP [21–23, 47]. In addition, most of the abraded zirconia specimens showed no wear crater and only slight surface scratches when examined under scanning electron microscopy using low magnification [21, 23, 47, 51]. Nevertheless, the grain dislodgment phenomenon accounts for the irregular pits that can be detected under high magnification, with larger pits seen in 5Y-TZP according to its larger grain size [47]. SEM images from Zhang et al. showed no crack formation in any worn zirconia specimens, particularly in 5Y-TZP which has a similar fracture toughness and strength to lithium disilicate [47]. In another study, a more aggressive wear pattern was observed in a comparative study by Vardhaman et al. Multilayered zirconia with a 5Y-TZP enamel layer and 4Y-TZP dentin layer was more susceptible to fatigue wear than monochromatic, monolithic 3Y-TZP. SEM evaluation also revealed a more advanced stage of wear progress in multilayered zirconia. Despite the variance in wear patterns, quantitative wear loss of the two materials suggested only minor differences [24].

It can be assumed that wear behavior is relatively unaffected by microstructural variations between zirconia generations and zirconia with increased yttria content is able to maintain good surface integrity despite lower flexural strength and fracture toughness [21, 23, 24, 47]. However, studies on the wear of unlayered first-generation zirconia are rare, since their occluding surfaces are usually layered with veneering porcelain. A summary of the studies on wear resistance of zirconia is depicted in Table 1.

### 3.3. Antagonist Wear against Zirconia

Wear resistance of zirconia compared to other restorative materials has been extensively studied. A wide range of antagonist materials have been used, including enamel substitutions, such as steatite ball or IPS Empress, bovine enamel, both nonstandardized and standardized human enamel, and zirconia itself. Studies on antagonist material wear against zirconia are listed in Table 2.

#### 3.3.1. Enamel Wear

*In vivo* studies on enamel wear opposing a monolithic zirconia crown found that average vertical wear of the enamel antagonist was considerably higher than the contralateral enamel controls at the same time interval [3, 74, 75]. A higher wear rate during the first 6 months suggested an initial running-in period followed by more steady wear afterward. Even though this rate was deemed higher than yearly physiologic enamel wear, evidence from other *in vitro* studies suggests that monolithic zirconia produced the least wear on enamel compared to other ceramics [71, 73, 83]. Furthermore, several studies have also stated minimal enamel wear when opposed to zirconia.

#### 3.3.2. Wear of Other Restorative Materials

The wear behavior of zirconia restorations opposed by dental materials other than human enamel is of equal significance. Unrestored opposing teeth are occasionally found. Comparative studies of various types of restorative materials when used against zirconia have also been performed [21, 50, 78–82].

*(1) Ceramics*. The *in vitro* wear rate of various types of ceramics opposing a zirconia abrader has been studied. The level of surface wear is associated with the materials' flexural strength, fracture toughness, and microstructure. Glass and hybrid ceramics consist of crystal grains loosely bound in glassy or polymeric matrices. According to the brittle nature of ceramics, the wear mechanism is primarily via fatigue wear [81]. Gradual wear of matrix phase exposes crystals to microfracture and dislodgment, thus lowering the strength of the material and increasing the chance of surface fracture and wear [21]. Furthermore, the wear rate of lithium-based glass ceramics, leucite-based ceramics, and feldspathic porcelains increases, respectively, inverse to their flexural strength [50]. According to the literature, selection of a glass ceramic restoration to oppose against a zirconia restoration may result in more substantial wear of the glass ceramic side [21, 78].

*(2) Metal*. Existing metal restorations in good condition may serve many years in function without the need for replacement. Caution must be exercised when a new zirconia restoration is contemplated being placed opposing an existing metal alloy restoration in terms of wear. Unlike ceramics, the wear mechanism of metal is associated with surface hardness and plastic deformation [4, 5]. The wear mechanism between metal alloys and zirconia is mainly abrasive wear, although some delamination wear was shown to occur in some circumstances [79]. Nevertheless, gold alloys show the greatest material loss as a consequence of their low hardness when abraded against monolithic zirconia, compared to nickel-chromium alloys and cobalt-chromium alloys, respectively [79]. Base metal alloys, with their greater surface hardness, are expected to possess higher wear resistance but more opposing antagonist wear [79]. SEM imaging reveals a roughened surface, surface cracking, and phase transformation of 3Y-TZP zirconia after abraded against cobalt-chromium alloys. Therefore, pairing of these two materials in a clinical setting is not recommended [79].

*(3) Polymer and Composite*. Use of different materials in full-arch implant-supported prostheses may be advocated to limit mechanical complications in the opposing, weaker arch. Accordingly, denture teeth supported by a metal framework are often chosen as an antagonist against a zirconia full-arch implant-supported prosthesis [84, 85].

The chemical compositions of resin matrix and filler in denture teeth influence their wear resistance. Modification in composition and fabrication technique has been used by manufacturers to improve the strength and wear resistance of conventional polymethyl methacrylate (PMMA) denture teeth. CAD/CAM-fabricated and 3D-printed denture teeth have shown comparable wear resistance to prefabricated PMMA denture teeth against a zirconia abrader [80, 82]. 3D-printed denture teeth often contain microbubble clusters under SEM inspection, which may initiate cracks after a functional load and period. Nevertheless, 3D-printed denture teeth remain relatively smooth after being abraded against zirconia, compared to the cracked and worn surfaces seen in worn prefabricated denture teeth [80]. PMMA denture teeth, regardless of their physical variations, show inferior wear resistance to resin composite denture teeth. This difference in wear resistance is consistent against both human enamel and zirconia [82, 86, 87]. A different wear mechanism has been described between unfilled polymer teeth, which were subjected to fatigue wear, and filled polymer or resin composite teeth that exhibit abrasive wear due to the presence of hard filler particles. Filled resin teeth provide superior wear resistance to unfilled ones under identical testing methodologies [80, 82].

The size and configuration of the filler particles themselves play an important role in the level of wear of resin composite. When abraded against a zirconia antagonist, microhybrid resin composite experienced higher volumetric substance loss as a consequence of its mixture of larger filler size and irregular filler shape compared to nanofilled resin composite.

## 4. Clinical Relevance

- Enamel wear against zirconia is considered clinically acceptable when zirconia is meticulously polished.
- In a situation where ceramic restorations are required on opposing occluding surfaces, zirconia placed against itself provides the best wear resistance, while choosing different types of ceramics can predispose the materials to greater wear.
- Placement of 3Y-TZP zirconia opposing a gold alloy restoration may result in increased wear on the gold alloy. On the contrary, placing zirconia against Co-Cr alloy is neither recommended since phase transformation is expected in 3Y-TZP zirconia.
- Filled composite resin denture teeth have improved wear resistance against 3Y-TZP zirconia restoration compared to unfilled PMMA denture teeth both prefabricated and CAD/CAM fabricated.

## 5. Conclusion

Based on this review of the literature, the following assumptions were drawn:

- Translucent zirconia compositions with increased yttria content (4Y- and 5Y-TZP), despite having different microstructural compositions, lower flexural strength, and lower fracture toughness, suggest equivalent wear resistance as conventional 3Y-TZP from the literature revised.
- Comparative studies evaluating wear of antagonist materials against zirconia compositions of different yttria content are still lacking. In the future, authors should be very specific in stating the zirconia composition used in scientific studies. Further studies regarding this issue are required.

## Figures and Tables

**Table 1:** Studies investigating wear resistance of zirconia specimens.

Authors	Yttria content [25]	Zirconia system	Zirconia specimen	Comparative groups	Antagonist material	Wear method: parameter	Results
Janyavula et al. [58]	3 mol% [25]	Ivoclar Vivadent	Flat shape	(1) Polished zirconia	Enamel (premolar cusp)	University of Alabama: 10 N load, frequency of 20 cycles/min, 2 mm distance, 400,000 cycles	Small amount of wear was observed in glazed and polished then glazed, while wear volume of polished zirconia specimens was unmeasurable.
				(2) Glazed zirconia			
				(3) Polished then glazed zirconia			

Jung et al. [71]	3 mol% [25]	Prettau	Cuboidal shape	(1) Zirconia	Enamel (maxillary premolar)	SD Mechatronik: 49 N load, 0.8 Hz frequency, 0.3 mm distance, 240,000 cycles	Volume loss of polished zirconia was the lowest among all groups presenting the best wear resistance. Glazed zirconia showed better wear resistance than feldspathic porcelain although not statistically different.
				(i) Polishing			
				(ii) Glazing			
				(2) Feldspathic porcelain			

Albashaireh et al. [50]	3 mol% [25]	IPS e.max ZirCAD	Disc shape	(1) Zirconia	Zirconia ball	Mastication simulator: 49 N load, 1.3 Hz frequency, 300,000 cycles	Zirconia specimens exhibited lowest vertical and volume loss compared to other ceramics after wear test. SEM images revealed no crack or defect on the surface of zirconia.
				(2) Lithium disilicate glass ceramic			
				(3) Leucite-reinforce glass ceramic			
				(4) Fluorapatite glass ceramic			
				(5) Nanofluorapatite glass ceramic			

Kwon et al. [72]	3 mol% [25]	Prettau	Anatomic crown (substrate), flat (antagonist)	(1) Enamel cusp	Zirconia	TE77 Auto: 50 N load, 1 Hz frequency, 15 mm distance, 600 cycles	(i) Enamel specimen wore at the highest rate against zirconia antagonist at the statistically different value from other two groups.
				(2) Gold alloy type 3			(ii) Gold alloy and zirconia represented similar wear resistance against zirconia.
				(3) Zirconia			(iii) The zirconia antagonists, however, were unblemished under SEM after tested with enamel and gold and presented only slight wear line against zirconia.

Nakashima et al. [73]	3 mol% [25]	Aadva Zr (GC)	Cone-shaped stylus	(1) Zirconia	Enamel (proximal surface of premolar)	University of Alabama: 75 N load, 1.2 Hz frequency, 100,000 cycles, back-and-forth rotating movement of 15°	Zirconia stylus showed very minimal wear which was substantially lower than other materials. Enamel antagonists opposing zirconia also presented with the lowest wear. Glass ceramics generated similar wear of enamel antagonist as the enamel stylus and also displayed similar wear resistance.
				(2) Lithium disilicate glass			
				(3) Leucite-reinforced glass			
				(4) Feldspathic porcelain			
				(5) Enamel cusp (molar)			

Kwon et al. [23]	3Y-TZP 5Y-ZP	Katana HT Katana UTML	Flat shape	(1) Polished zirconia	Enamel cusp (mandibular molars)	University of Alabama: 20 N load, 0.4 Hz frequency, 2 mm distance, 300,000 cycles	Both 3Y- and 5Y-TZP zirconia specimens presented comparable unmeasurable wear volume, while another two groups displayed significant wear. SEM images showed neither surface fracture nor roughening of any zirconia surface. However, opposing enamel cusps showed no difference among material groups.
				(i) Katana HT (3Y-TZP)			
				(ii) Katana UTML (5Y-ZP)			
				(2) Lithium disilicate			
				(3) Enamel (labial surface of maxillary central incisor)			

Borrero-Lopez et al. [21]	3Y-TZP 5Y-TZP Graded	Zpex Zpex Smile Zpex (graded)	Disc shape	(1) Zirconia	Densely sintered zirconia ball (3Y-TZP)	Rotating ball-on-3-flat tribometer: 30 N load, 30 rpm frequency, total contact distance of 37 m	(i) All types of zirconia specimens showed the similar lowest wear rate when tested against 3Y-TZP zirconia, which were lower than other test groups.
				(i) Zpex (3Y-TZP)			(ii) SEM images revealed noticeable scratch marks on the surface of zirconia although no wear scar was observed, unlike other materials where obvious wear scars were presented.
				(ii) Zpex Smile (5Y-TZP)			
				(iii) Zpex (graded)			
				(2) Lithium disilicate			
				(3) Feldspathic ceramic			
				(4) Ceramic-polymer composites			
				(5) Enamel			

Vardhaman et al. [24]	4Y-, 5Y-TZP 3Y-TZP	IPS e.max ZirCAD Multi IPS e.max ZirCAD LT	Flat shape (substrate) Spherical shape (antagonist)	(1) Multilayered zirconia	Zirconia	OHSU oral wear simulator: 30 N load, 1.5 Hz frequency, 5 mm contact distance, maximum of 50,000 cycles	(i) Multilayered zirconia showed greater volume loss and deeper wear depth after simulation. Furthermore, wear pattern of this group was more aggressive with subsurface fracture.
				(i) Enamel layer: 5Y-TZP			(ii) Zirconia antagonist revealed unmeasurable wear scars for both groups.
				(ii) 2 transition layers			
				(iii) Dentin layer: 4Y-TZP			
				(2) 3Y-TZP zirconia			

Rosentritt et al. [22]	3Y-TZP 4Y-TZP 5Y-TZP	DD Bio ZX2 DD cube ONE DD cubeX2	Disc shape (substrate)	(1) 3Y-TZP	Steatite ball	Pin-on-block wear test: 50 N load, 1.2 Hz frequency, 1 mm contact, 120,000 cycles	(i) All zirconia showed comparable wear behavior in either material wear or antagonist wear.
				(2) 4Y-TZP			(ii) Lithium disilicate group exhibited greater material wear but lower antagonist wear compared to all zirconia.
				(3) 5Y-TZP			
				(4) Lithium disilicate			
				(5) Enamel			

^*∗*^Information on yttria content, which is not available in the original literature, is listed according to the review literature by Kontonasaki et al. [25]. Studies are listed in chronological order.

**Table 2:** Studies investigating antagonist wear against zirconia specimens.

Authors	Yttria content [25]	Zirconia system	Zirconia specimen configuration	Comparative groups	Antagonist material	Wear method: parameter	Results
Albashaireh et al. [50]	3 mol% [25]	IPS e.max ZirCAD	Disc shape	(1) Zirconia	Zirconia ball	Mastication simulator: 49 N load, 1.3 Hz frequency, 300,000 cycles	Zirconia specimens exhibited lowest vertical and volume loss compared to other ceramics after wear test. SEM images revealed no crack or defect on the surface of zirconia.
				(2) Lithium disilicate glass ceramic			
				(3) Leucite-reinforce glass ceramic			
				(4) Fluorapatite glass ceramic			
				(5) Nanofluorapatite glass ceramic			

Preis et al. [51]	4.5–5.4 wt.% 4.5–5.4 wt.% N/A 4.5–6 wt.% 5 wt.%	(i) Ceramill ZI (ii) Digizon (iii) Lava (iv) Zeno Zr Bridge (v) Cercon base	Disc shape	(1) Monolithic zirconia	(1) Steatite balls (2) Enamel	EGO chewing simulator: 50 N load, 1.6 Hz frequency, 1 mm contact distance, 120,000 cycles	(i) None of the monolithic zirconia specimens displayed distinctive wear crater, only surface scratches, for both steatite and enamel antagonists.
				(i) Ceramill ZI			
				(ii) Digizon			
				(iii) Lava			
				(iv) Zeno Zr Bridge			
				(v) Cercon base			(ii) Veneered zirconia groups showed similar significant vertical substance loss. SEM images revealed apparent plowed surface after glaze layer worn off.
				(2) Veneered zirconia			
				(i) Cercon base (polished-veneered)			
				(ii) Cercon base (sandblasted-veneered)			
				(3) Feldspathic porcelain			
				(i) Cercon Ceram Kiss			(iii) Vertical loss of all feldspathic groups were considerably higher than zirconia groups, but lower than those of enamel control.
				(ii) Creation Zi-F			
				(iii) Lava Ceram			
				(iv) VITA OMEGA 900			
				(4) Enamel			

Kim et al. [48]	3 mol% [25] 3 mol% [25] N/A	Prettau Lava Rainbow	Cuboidal shape	(1) Zirconia	(i) Enamel cusp (premolar)	SD Mechatronik: 49 N load, thermocycling 5/55°C	Antagonist wear against 3 types of zirconia was significantly lower than lithium disilicate and feldspathic ceramic, respectively.
				(i) Prettau (3Y-TZP)			
				(ii) Lava (3Y-TZP)			
				(iii) Rainbow (3Y-TZP)	(ii) Feldspathic porcelain cusp		
				(2) Lithium disilicate			
				(3) Feldspathic porcelain			

Park et al. [62]	3 mol% [25] N/A 3 mol% [25]	Prettau ZirBlank Zeno Zr	Disc shape	(1) Prettau—polished	Enamel cusp (maxillary premolar)	SD Mechatronik: 49 N load, 1.3 Hz frequency, 0.3 mm distance, 240,000 cycles	(i) The highest volume loss of enamel antagonist was presented in the feldspathic group.
				(2) Prettau—polished and stained			
				(3) Prettau—stained and glazed			
				(4) ZirBlank			(ii) Surface treatment method influenced wear of antagonist with the lowest value by polished zirconia, followed by stained and stained then glazed zirconia.
				(5) Zeno Zr			
				(6) Feldspathic porcelain			

Stober et al. [74]	3 mol%	Zenostar Zr Translucent	Anatomic crown (molars)	(1) Zirconia	Enamel	*In vivo*: 6-month follow-up	Zirconia crown related to higher enamel wear than natural enamel opposing each other.
				(2) Enamel (contralateral side)			

Mundhe et al. [3]	3 mol% [25]	Lava	Anatomic crown (mandibular first molar)	(1) Zirconia crown	Enamel (maxillary premolar and molar)	*In vivo*: 1-year follow-up	(i) Enamel wear opposing zirconia crown was lesser than metal-ceramic crown, but higher than natural enamel.
				(2) Metal-ceramic crown			

Kwon et al. [72]	3 mol% [25]	Prettau	Anatomic crown (substrate), flat (antagonist)	(1) Enamel cusp	Zirconia	TE77 Auto: 50 N load, 1 Hz frequency, 15 mm distance, 600 cycles	(i) Enamel specimen wore at the highest rate against zirconia antagonist at the statistically different value from other two groups.
				(2) Gold alloy type 3			(ii) Gold alloy and zirconia represented similar wear resistance against zirconia.
				(3) Zirconia			(iii) The zirconia antagonists, however, were unblemished under SEM after tested with enamel and gold and presented only slight wear line against zirconia.

Stober et al. [75]	3 mol% ^*∗*^	Zenostar Zr Translucent	Crown	(1) Zirconia	Enamel	*In vivo*: 12-, 24-month follow-up	(i) A continuous study of Stober et al. (2014) at one- and two-year interval.
				(2) Enamel (contralateral side)			(ii) Zirconia crown generated greater wear rate during initial run-in period and decelerated afterwards. The amount of enamel antagonist wear produced by zirconia crown was twice that of natural enamel.

Stawarczyk et al. [17]	3 mol% 3 mol% 3 mol% 3 mol% 3 mol% [25]	Zenostar DD Bio ZX [1] Ceramill Zolid inCoris TZI Ceramill ZI	Disc shape	(1) Second-generation zirconia	Enamel (molar cusp)	SD Mechatronik: 50 N load, 1 Hz frequency, 0.7 mm distance, maximum of 1,200,000 cycles	(i) First-generation zirconia with veneering porcelain showed the highest material loss for both zirconia itself and enamel antagonist.
				(i) Zenostar			(ii) In second-generation monolithic zirconia groups, all glazed groups showed higher material and enamel loss than polished groups. There was no difference in material wear among the polished zirconia groups.
				(ii) DD Bio ZX2			
				(iii) Ceramill Zolid			
				(iv) inCoris TZI			(iii) Antagonist wear was brand dependent.
				^*∗*^Each group has 2 subgroups			
				(i) Polished zirconia			
				(ii) Glazed zirconia			
				(2) First-generation zirconia			
				(i) Ceramill ZI (veneered)			

Nakashima et al. [73]	3 mol% [25]	Aadva (GC)	Rounded-tip cone shape	(1) Zirconia	Enamel (proximal surface of premolar)	University of Alabama: 75 N load, 1.2 Hz frequency, back-and-forth rotating movement of 15°, 100,000 cycles	(i) Zirconia stylus showed very minimal wear which was substantially lower than other materials.
				(2) Lithium disilicate glass			(ii) Enamel antagonists opposing zirconia also presented with the lowest wear.
				(3) Leucite-reinforced glass			
				(4) Feldspathic porcelain			(iii) Glass ceramics generated similar wear of enamel antagonist as the enamel stylus and also displayed similar wear resistance.
				(5) Enamel cusp (molar)			

Lohbauer and Reich [76]	3 mol% [25]	Lava Plus	Anatomic crown (premolar-molar)	(1) Zirconia	Enamel	*In vivo*: 2-year follow-up	No difference in material loss was observed.
				(2) Enamel			

Yang et al. [77]	4-5 wt.% 7.12–7.16 wt.%	Rainbow Katana ML	Cuboidal shape	(1) Zirconia	Enamel (premolar cusp)	SD Mechatronik: 49 N load, 1.5 Hz frequency, 2 mm contact distance, 100,000 cycles	(i) Zirconia abraded opposing enamel more than natural enamel.
				(i) Rainbow			
				(ii) Katana ML			(ii) Wear resistance of zirconia was brand dependent.
				(2) Enamel (flat surface of premolar)			

Borrero-Lopez et al. [21]	3Y-TZP 5Y-TZP Graded	Zpex Zpex Smile Zpex (graded)	Disc shape	(1) Zirconia	Densely sintered zirconia ball (3Y-TZP)	Rotating ball-on-3-flat tribometer: 30 N load, 30 rpm frequency, total contact distance of 37 m	All types of zirconia specimens showed the similar lowest wear rate when tested against 3Y-TZP zirconia, which were lower than other test groups. SEM images revealed noticeable scratch marks on the surface of zirconia although no wear scar was observed, unlike other materials where obvious wear scars were presented.
				(i) Zpex (3Y-TZP)			
				(ii) Zpex Smile (5Y-TZP)			
				(iii) Zpex (graded)			
				(2) Lithium disilicate			
				(3) Feldspathic ceramic			
				(4) Ceramic-polymer composites			
				(5) Enamel			

Hayashi et al. [78]	5.2 mass%	Zpex100	Flat (substrate), hemispherical shape (antagonist)	(1) Bovine enamel	Zirconia	Wear simulator: 10 N load, 90 cycles/min velocity, 3 mm distance, 30,000 cycles	(i) Zirconia specimens against zirconia antagonist showed excellent wear resistance with unmeasurable wear volume.
				(2). Resin composite: hybrid filler			
				(3) Resin composite: nanofiller			(ii) Glass ceramic left obvious wear track and greater wear volume than other materials with lithium disilicate higher than porcelain, followed by hybrid resin composite, bovine enamel, and nanofilled resin composite.
				(4) Feldspathic porcelain			
				(5) Lithium disilicate glass			(iii) Zirconia abraders were roughened from glass ceramic specimen but not from softer resin composite.
				(6) Zirconia: Zpex100			

Cha et al. [79]	5.3 mass% (3Y-TZP)	Zpex	Disc shape	(1) Co-Cr alloy	Zirconia	SD Mechatronik: 49 N load, 1 mm distance, 240,000 cycles	(i) Gold alloy specimens presented the highest wear volume loss, followed by Ni-Cr alloy and Co-Cr alloy.
				(2) Ni-Cr alloy	With 2 subgroups (1) Smooth surface (2) Rough surface		(ii) Rough surface zirconia exacerbated wear on all alloys.
				(3) Gold alloy			(iii) Co-Cr alloys, although presented good wear resistance, roughened zirconia antagonists and induced surface crack and phase transformation after abrasion. Therefore, clinical application of Co-Cr alloy opposing zirconia was not recommended.

Cha et al. [80]	N/A	ZirPremium UT+	Hemispherical shape	(1) Prefabricated PMMA	Zirconia Co-Cr alloy	SD Mechatronik: 49 N load, 2 mm contact distance, 30,000 cycles, 5°/55°C thermocycling	(i) 3D-printing denture teeth showed comparable wear resistance to prefabricated PMMA.
				(2) Prefabricated cross-linked PMMA			
				(3) 3D-printing MA-based			
				(4) Prefabricated MPM-PMMA, hybrid fillers			(ii) Zirconia antagonist produced smoother wear surface compared to Co-Cr alloy that generated surface cracks.
				(5) Prefabricated CLC-PMMA-OFC, microfillers			

Wedler et al. [81]	3Y-TZP	3Y-TZP zirconia	Spherical shape	(1) Lithium disilicate:	Zirconia	Elf-3300 electrodynamic biaxial mouth-motion simulator: 200 N load, 1.5–2.0 Hz frequency, maximum of 1 million cycles	(i) Rate of wear process was varied among materials depending on their wear mechanism.
				(i) IPS e.max CAD			(ii) Glass ceramics exhibited lower wear rate which is primarily based on fatigue wear due to its brittle nature.
				(ii) Suprinity PC			(iii) Nanoparticle resin composite wore through abrasive wear, resulting in larger wear crater.
				(2) Feldspar-reinforced aluminosilicate glass: VITABLOCS MARK II			(iv) PIRGN presented combination of two wear mechanisms caused greater wear rate from the synergistic effect.
				(3) Polymer-infiltrated reinforced-glass network (PIRGN): Enamic			
				(4) Prepolymerized nanoparticle resin composite: Lava Ultimate			

Esquivel et al. [82]	5–8 mass% ^*∗*^	Katana LT	Cone shape	(1) Cross-linked PMMA	Zirconia	Custom dual-axis mastication simulator: 20 N load, 1 Hz frequency, 2 mm contact distance, 200,000 cycles	(i) Nanohybrid composite resin teeth showed the best wear resistance against zirconia.
				(2) Cross-linked acrylate polymer (CAD/CAM)			
				(3) Cross-linked PMMA (CAD/CAM)			(ii) Small differences were found among polymer teeth groups, ranking from the highest in group 1 to the lowest in group 3, respectively.
				(4) Nanohybrid composite resin			

^*∗*^Information on yttria content, which is not available in the original literature, is listed according to the review literature by Kontonasaki et al. [25] and acquired by personal contact with the manufacturers. N/A, no information available. Studies are listed in chronological order.

## Data Availability

The data used to support the findings of this study are included within the article.
